# Supplementary material for: Functional and structural reorganization in brain tumors: a machine learning approach using desynchronized functional oscillations
Source: Commun Biol. 2024 Apr 6;7:419. doi: 10.1038/s42003-024-06119-3 (PMC10998892; doi:10.1038/s42003-024-06119-3)
Supplement: Supplementary file 2 — Supplementary Material [file 42003_2024_6119_MOESM2_ESM.pdf]

Supplementary Material

Functional and Structural Reorganization in Brain Tumors: A Machine Learning Approach Using Desynchronized Functional Oscillations

Joan Falcó-Roget[1,*], Alberto Cacciola[2], Fabio Sambataro[3], and Alessandro Crimi[1,4,*]
[1] Brain and More Lab, Computer Vision, Sano Centre for Computational Medicine, Kraków, Poland
[2] Brain Mapping Lab, Department of Biomedical, Dental Sciences and Morphological and Functional Images, University of Messina, Messina, Italy
[3] Department of Neuroscience, University of Padova, Padua, Italy
[4] Faculty of Computer Science, AGH University of Krakow, Kraków, Poland
[*] Corresponding Author at: j.roget@sanoscience.org a.crimi@sanoscience.org

Padding and truncating Fourier transforms to solve the TR inconsistency between fMRI scans

Functional MRI samples neural signal every a specific number of seconds. The time between two volume acquisitions, known as the repetition time (TR), is usually kept constant throughout the study. As already stated in the main text, the TR was accidentally changed from 2100 ms to 2400 ms. The effects of acquiring fMRI with different TRs have been studied for a considerable amount of time (see for example[1,2]). Briefly, longer TRs offer better signal-to-noise ratios (SNRs) and are not recommended for fixed time paradigms (i.e., scans of fixed duration). Furthermore, the number of volumes should be first selected and then be kept constant throughout the experiment. Therefore, resting-state data offers more reliable SNRs mainly due to the need of rapid sampling during task conditions[3]. The dataset we studied contained resting-state scans with relatively long TRs. Most importantly, after the accidental TR shift, the time of acquisition was not kept fixed (see Methods). Hence, it resulted in slightly longer series with the same number of volumes.

Nonetheless, the discrepancy between subjects needed to be addressed accordingly. Recall that we based our technique on discrete Fourier decompositions where the properties rely on the sampling frequencies and the sampling rates[1]. Given a TR and a signal of length n, the difference between two consecutive Fourier sampling frequencies is

$$\Delta\omega_{\text{sampled}} = \frac{1}{\text{TR} \cdot n},$$

a ratio which should be preserved even in the case of having two different sampling rates or TRs. This simple heuristic yields a relationship between the number of points of the two signals: the ratio between n and the TR should be constant. Therefore, series with shorter TRs were extended Δn points by 0-padding them, with

$$\Delta n = n\left(\frac{\text{TR}_1 - \text{TR}_2}{\text{TR}_2}\right) \qquad (S1)$$

where $\text{TR}_1 > \text{TR}_2$ and 1 extra point is added after ensuring that the division outputs an integer number. After computing the transformation, the resulting frequency spectrum did not share the same length. Consequently, the spectrum from the shorter series was simply

[1] Fourier Transforms (scipy.fft)

truncated at the same length as the rest of the spectrums. After inspecting the original spectrums (see Fig. 1; see also Fig. S2-S5) we noticed that no power was allocated to high frequencies thus ensuring no power loss after the truncation. We provide a comprehensive example with a sinusoidal signal consisting of only two frequencies in Fig. S14.

Noteworthy, the total power of the signal after the extension is not kept constant, but the distribution of the power is correctly maintained (Fig. S14). Since the DAS defined in the text does not care about absolute power but rather their distributions, this approach safely preserved information of the series' dynamics. However, all the absolute power analyses conducted in this study were done considering the unpadded signals – although the statistical results were identical to those ones obtained with the padded and truncated signals (i.e., up to the third decimal point).

Generation of post-surgery graphs with artificial neural networks

A connectome for the n-th subject at a particular time point t which, in our case, was mapped to control, pre- and post-surgery stages t_c, t_{pre} and t_{post}. Only the lower triangular part of the connectomes was flattened into a vector $\boldsymbol{x}_n(t)$ of E components, given that structural connections are always symmetric and no self-loops were considered. Each one of these components $x_{nk}(t)$ with $k = 1, \dots, E$ represents a link of strength ϵ between two brain regions. We simplify the notation and refer to pre-surgery connectomes as $\boldsymbol{x}_n$, to post-surgery connectomes as $\boldsymbol{y}_n$ and to healthy connectomes as $\boldsymbol{z}_n$.

Following previous work[4], we defined a distribution of binary links based on connectomes from N_C healthy subjects. For the k-th edge, $\lambda_k = 0{,}1$ is a Bernoulli distributed binary variable with probability

$$P(\lambda_k = 1) = \frac{1}{N_c} \sum_{n=1}^{N_c} \Theta(z_{nk} - \theta) \qquad (S2)$$

where $\Theta(\cdot)$ is the Heaviside step function and $\theta = 0.2$ is a threshold that filters links with insufficient strength and therefore minimizing the false positive rate. For the choice of the threshold, we considered the Shannon entropy and the modularity[5,6] of the network built according to Eq. (S2). We observed that small thresholds maintained a high entropy allowing for the inclusion of more connections without excessively altering the structure of the prior (Fig. S7). Lower thresholds allow more variability in the generated prior since more spurious connections can be considered.

For each patient and edge, the post-surgery probability of having a meaningful link $\lambda_k = 1$ with strength ϵ is conditioned on the pre-operative graph. We exploit this fact with the well-known Bayes' theorem,

$$P(y_{nk} = \epsilon, \lambda_k = 1 \mid \boldsymbol{x}_n) = \mathcal{L}(y_{nk} = \epsilon \mid \boldsymbol{x}_n)\, P(\lambda_k = 1) \qquad (S3)$$

where $\mathcal{L}(y_{nk} = \epsilon \mid \boldsymbol{x}_n)$ is the likelihood of having a post-surgery ϵ-strengthened connection conditioned on the pre-surgery connectome. For Eq. (S3) to hold, $P(\lambda_k = 1)$ must be independent on the pre-surgery graph $\boldsymbol{x}_n$. This assumption is reasonable since the anatomical prior was built using only healthy controls, therefore, not considering lesioned connections. We sampled each k-th connection using the Maximum A Posteriori criterion to generate the post-surgery graphs. To train the network, the sampled connections were then compared to the ground truth using the Mean Squared Error (MSE).

Although many possibilities emerge for estimating the likelihood in Eq. (S3), we used a fully connected network with one hidden layer of size $E/2$. Therefore, for each edge k in the n-th connectome, the likelihood in Eq. (S3) was obtained by propagating the input through the artificial network as follows:

$$\begin{cases} r_{ni} = \sigma\left(\sum_{j=1}^{E} W_{ij}^{\text{input}} \cdot x_{nj}\right) \\ \mathcal{L}(y_{nk} = \epsilon \mid \boldsymbol{x}_n) = \sum_{i=1}^{E/2} W_{ki}^{\text{output}} \cdot r_{nj} \end{cases}$$

where $\sigma(x) = \frac{1}{1+e^{-x}} \in [0,1]$ is a sigmoid activation function. Note how the network compresses the information in the intermediate layer forcing a low dimensional representation of connectomes' features. The model weights W_{ij}^{input} and W_{ij}^{output} were updated using stochastic gradient descent to minimize the mean squared error between the connectomes generated by the model and the ones obtained by applying the tractography pipeline described in the main text. Several architectures were considered, but we found that adding more layers or even considering 1D or 2D convolutions did not add significant improvements to the model. We report the mean results for a two hidden layer fully connected layer in Table S2.

Weight probability distribution of brain networks

For all graphs, we computed the degree of each node by summing over all the connections

$$D_i = \sum_{k=1}^{N} \omega_{ik}, \tag{S4}$$

where N is the number of nodes, 166 in our graphs. To compute the probability distribution of a node having degree d, we counted all the nodes whose degree fell in a bin of width $\Delta\omega$ as

$$P(d = D) = \frac{1}{N} \sum_{D_i \in [d, d+\Delta\omega]} D_i, \tag{S5}$$

where N is again the number of nodes that acts as a normalization constant.

Networks wired at random have a binomial degree distribution, however it was shown that biological networks deviate from this tendency towards a scale-free or, in the specific case of brain networks, a broad-scale distributions[7,8]. In both cases, the number of highly connected nodes (i.e., nodes with large degree) decays following a power-law in the case of scale-free and an exponentially truncated power-law for broad-scale distributions. However, for weighted networks, it has been shown that a lognormal distribution is better suited to describe brain connections[9,10].The probability density of a lognormally distributed variable follows

$$P(W = \omega) = \frac{1}{\omega\sigma\sqrt{2\pi}} e^{-\frac{(\ln \omega - \mu)^2}{2\sigma^2}} \tag{S6}$$

where μ, σ are the geometric mean and standard deviation. Note, that the main manuscript deals with the variable $1 + \omega$, therefore Eq. (S6) should be corrected for this. Given that the Jacobian of the transformation is 1, the proof is straightforward.

$$P(W = 1 + \omega) = \frac{1}{(\omega - 1)\sigma\sqrt{2\pi}} e^{-\frac{(\ln[\omega-1]-\mu)^2}{2\sigma^2}} \tag{S7}$$

Therefore, the variable $1 + \omega$ also follows a lognormal distribution as seen in Fig. 6 of the main text.

We adjusted a power law distribution to the weighted degrees[11],

$$P(x) \propto x^{-\alpha}, \tag{S8}$$

where α is the power law exponent measuring the decay in the probability of finding strongly connected nodes in the network (e.g., hubs). We independently fit a power law distribution to each network derived from the healthy cohort and patient. Importantly, for each patient, we fit a power law distribution for the network obtained with our pipeline and another fit for the network obtained with a canonical multi-shell multi-tissue approach without masking the tumor. Next, we compared the differences between the exponent α for each pipeline and the healthy cohort and the differences between pipelines (see Fig. S6d). Importantly, we consider two different scaling ranges: $x_{min} = 100$ and $x_{min} = 300$, the latter one evaluating the scale-freeness in the large asymptotic range.

Subject specific results and model comparison

Subject	MSE	MAE	PCC	CS	KL	JS
sub-PAT25	2.173	0.869	0.774	0.829	9.117	0.684
sub-PAT05	2.047	0.870	0.784	0.842	9.443	0.699
sub-PAT11	1.922	0.821	0.790	0.837	8.712	0.678
sub-PAT28	1.727	0.803	0.853	0.899	**8.150**	**0.655**
sub-PAT08	1.975	0.840	0.798	0.850	8.483	0.670
sub-PAT15	1.799	0.804	0.824	0.873	8.541	0.669
sub-PAT17	1.691	0.779	0.828	0.871	9.510	0.704
sub-PAT07	2.104	0.869	0.781	0.840	10.106	0.720
sub-PAT24	1.542	**0.747**	0.857	0.900	9.197	0.695
sub-PAT06	1.828	0.824	0.815	0.869	9.556	0.691
sub-PAT02	1.906	0.822	0.796	0.839	8.870	0.684
sub-PAT10	1.711	0.789	0.828	0.877	8.531	0.666
sub-PAT01	1.809	0.823	0.837	0.887	9.663	0.715
sub-PAT26	**1.523**	0.753	**0.869**	**0.910**	9.275	0.697
sub-PAT03	1.725	0.788	0.819	0.862	9.581	0.711
sub-PAT20	2.984	1.057	0.707	0.791	9.129	0.686
sub-PAT23	1.888	0.807	0.797	0.847	9.454	0.714
sub-PAT13	2.048	0.856	0.782	0.830	9.346	0.697
sub-PAT16	2.072	0.879	0.798	0.857	8.161	0.660

Table S1 Leave One Out cross validation results and a hybrid fiber reconstruction pipeline. Results for the tested subject in each trained model. (MSE: Mean Squared Error; MAE: Mean Absolut Error; PCC: Pearson Correlation Coefficient; CS: Cosine Similarity; KL Kullback-Leiber and JS: Jensen-Shannon Divergences). For each fold the subject tested was not included in the training set (see Methods). The best subject in each according to each metric is highlighted in bold. Correlations between all pairs of metrics were high (|r|>0.8 and p<0.01). Only in PCC and CS a higher number means a better fit. Bold numbers indicate better performance; lower is better for MSE, MAE, KL, and JS while higher is better for CS and PCC.

Model	MSE	MAE	PCC	CS	KL	JS
FCNET	**1.91 $\pm$ 0.07**	0.832 $\pm$ 0.015	**0.807 $\pm$ 0.008**	**0.859 $\pm$ 0.006**	**9.09 $\pm$ 0.1**	**0.681 $\pm$ 0.004**
2-layer	1.92 $\pm$ 0.08	**0.827 $\pm$ 0.016**	**0.807 $\pm$ 0.009**	0.858 $\pm$ 0.007	9.44 $\pm$ 0.11	0.699$\pm$ 0.004
FCNET (*msmt)*	1.92 $\pm$ 0.07	0.831 $\pm$ 0.014	0.808 $\pm$ 0.007	**0.859 $\pm$ 0.006**	9.43$\pm$ 0.10	0.699 $\pm$ 0.003

Table S2 Comparison of alternative models (mean $\pm$ SEM). Comparisson of alternative models using a Leave One Out cross validation scheme in 6 metrics (MSE: Mean Squared Error; MAE: Mean Absolut Error; PCC: Pearson Correlation Coefficient; CS: Cosine Similarity; KL Kullback-Leiber and JS: Jensen-Shannon Divergences). The last row corresponds to the same FCNET model but using as inputs brain networks obtained with the alternative multi-shell multi-tissue fiber reconstruction pipeline

(see Main Text; see also Methods). Bold numbers indicate better performance; lower is better for MSE, MAE, KL, and JS while higher is better for CS and PCC.

Subject	MSE	MAE	PCC	CS	KL	JS
sub-PAT25	2.161	0.858	0.774	0.829	9.024	0.679
sub-PAT05	2.051	0.860	0.783	0.841	9.772	0.711
sub-PAT11	1.846	0.809	0.799	0.843	8.889	0.683
sub-PAT28	**1.680**	0.796	0.854	**0.900**	9.842	0.713
sub-PAT08	1.838	0.820	0.813	0.862	8.559	**0.674**
sub-PAT15	1.775	0.794	0.828	0.876	9.434	0.696
sub-PAT17	1.683	0.779	0.829	0.872	9.189	0.690
sub-PAT07	2.161	0.883	0.775	0.835	9.697	0.704
sub-PAT24	1.619	**0.766**	0.849	0.894	9.363	0.694
sub-PAT06	1.937	0.848	0.803	0.861	9.494	0.698
sub-PAT02	1.736	0.791	0.819	0.856	9.915	0.715
sub-PAT10	1.767	0.799	0.823	0.873	9.977	0.713
sub-PAT01	1.921	0.845	0.827	0.880	10.290	0.725
sub-PAT26	1.709	0.791	**0.855**	**0.900**	9.201	0.698
sub-PAT03	1.685	0.776	0.823	0.865	9.650	0.709
sub-PAT20	2.888	1.042	0.717	0.798	8.985	0.691
sub-PAT23	1.875	0.807	0.796	0.847	8.955	0.694
sub-PAT13	1.863	0.821	0.802	0.845	**9.018**	0.683
sub-PAT16	2.219	0.909	0.782	0.846	10.011	0.722

Table S3 Leave One Out cross validation results and a multi-shell multi-tissue fiber reconstruction pipeline. Results for the tested subject in each trained model when using only a multi-shell multi-tissue fiber reconstruction method. (MSE: Mean Squared Error; MAE: Mean Absolut Error; PCC: Pearson Correlation Coefficient; CS: Cosine Similarity; KL Kullback-Leiber and JS: Jensen-Shannon Divergences). For each fold the subject tested was not included in the training set (see Methods). The best subject in each according to each metric is highlighted in bold. Correlations between all pairs of metrics were high (|r|>0.8 and p<0.01). Only in PCC and CS a higher number means a better fit. Bold numbers indicate better performance; lower is better for MSE, MAE, KL, and JS while higher is better for CS and PCC.

Quantifying the effects of the Repetition Time (TR) on the reorganization of the functional networks

To further understand the effects that the inconsistent TRs might have introduced to the analyses carried in this work we studied the same scores after excluding the 7 patients and 4 controls with TR=2.1s. While these types of procedures may effectively reduce the statistical power of the analyses, they can help to elucidate hidden confounders in the data. The reduced group showed a medium correlation between the network similarity measure and the DAS of the DMN (r=-0.215, p=0.392, df=18, two-tailed exact test) and a strong correlation between the alterations in the lesion and DMN (r=-0.603, p=0.008, df=18, two-tailed exact test).

We also did permutation analyses to determine whether the 7 excluded subjects with brain tumors represented a unique subsample or rather a random instantiation of 7 patients. We computed $N = 1000$ correlation coefficients between the same three magnitudes mentioned in the previous paragraph and in the main text after randomly excluding 7 patients from the pool. The exclusion was done by resampling without replacement from the patients' ID. For the network similarity analyses the effect of the TR appeared to be significant although it didn't alter the interpretation nor the conclusion of the main analyses (Fig. S15a). For the

relationship between local and global alterations in the DMN, the TR did not have a significant effect (Fig. S15b).

To exclude the possibility of reporting false inferences, we measured the exact contribution of the TR in network similarity measure. For that, we fitted two linear models that explained the differences in the Pearson correlation between the networks of the patients and controls as a function of the DAS and the TR:

$$\text{Node similarity} = a \cdot DAS + \text{intercept}, \quad (S9)$$

$$\text{Node similarity} = a \cdot DAS + b \cdot TR + \text{intercept}. \quad (S10)$$

Importantly, this analysis was performed with the whole dataset. For the first model in Eq. S9, which only included the DAS, the effect was, as expected, significant (a=-0.057, p=0.01, df=23, two-tailed t-test). The coefficient of determination was identical to the one in Fig. 2a; again, as expected. However, when explicitly including the TR, the coefficient in Eq. S10 quantifying the contribution of the TR to the node similarity measure was non-significant (b=0.178, p=0.139, df=22, two-tailed exact-test, CI=[-0.062,0.418]).

These results can be interpreted in the following way. The padding strategy correctly removed all the effects of the TR for the analyses that were performed solely on the frequency domain (e.g., local and global alterations). Yet, for the other case, although the contribution of the TR to the results was proven to be non-critical, the TR inconsistency remained slightly observable. However, and most importantly, all the interpretations and conclusions derived from the analyses in the main text remained identical and only decreased in size, as was expected given the reduced statistical power (Fig. S15c-f).

References

1. Constable, R. T. & Spencer, D. D., Repetition time in echo planar functional MRI. *Magnetic Resonance in Medicine: An Official Journal of the International Society for Magnetic Resonance in Medicine* **46**, 748–755 (2001).
2. Murphy, K., Bodurka, J. & Bandettini, P. A., How long to scan? The relationship between fMRI temporal signal to noise ratio and necessary scan duration. *Neuroimage* **34**, 565–574 (2007).
3. Welvaert, M. & Rosseel, Y., On the definition of signal-to-noise ratio and contrast-to-noise ratio for fMRI data. *PloS one* **8**, e77089 (2013).
4. Falcó-Roget, J. & Crimi, A., *Bayesian Filtered Generation of Post-surgical Brain Connectomes on Tumor Patients*, presented at MICCAI Workshop on Imaging Systems for GI Endoscopy, International Workshop on Graphs in Biomedical Image Analysis, 2022 (unpublished).
5. Blondel, V. D., Guillaume, J.-L., Lambiotte, R. & Lefebvre, E., Fast unfolding of communities in large networks. *Journal of statistical mechanics: theory and experiment* **2008**, P10008 (2008).
6. Newman, M. E. J., Modularity and community structure in networks. *Proceedings of the national academy of sciences* **103**, 8577–8582 (2006).
7. Barabási, A.-L. & Albert, R., Emergence of scaling in random networks. *science* **286**, 509–512 (1999).
8. Amaral, L. A. N., Scala, A., Barthelemy, M. & Stanley, H. E., Classes of small-world networks. *Proceedings of the national academy of sciences* **97**, 11149–11152 (2000).
9. Ercsey-Ravasz, M. *et al.*, A predictive network model of cerebral cortical connectivity based on a distance rule. *Neuron* **80**, 184–197 (2013).
10. Fornito, A., Zalesky, A. & Bullmore, E., *Fundamentals of brain network analysis* (Academic Press, 2016).

11. Alstott, J., Bullmore, E. & Plenz, D., powerlaw: A Python Package for Analysis of Heavy-Tailed Distributions. *PLoS ONE* **9**, e85777 (2014).

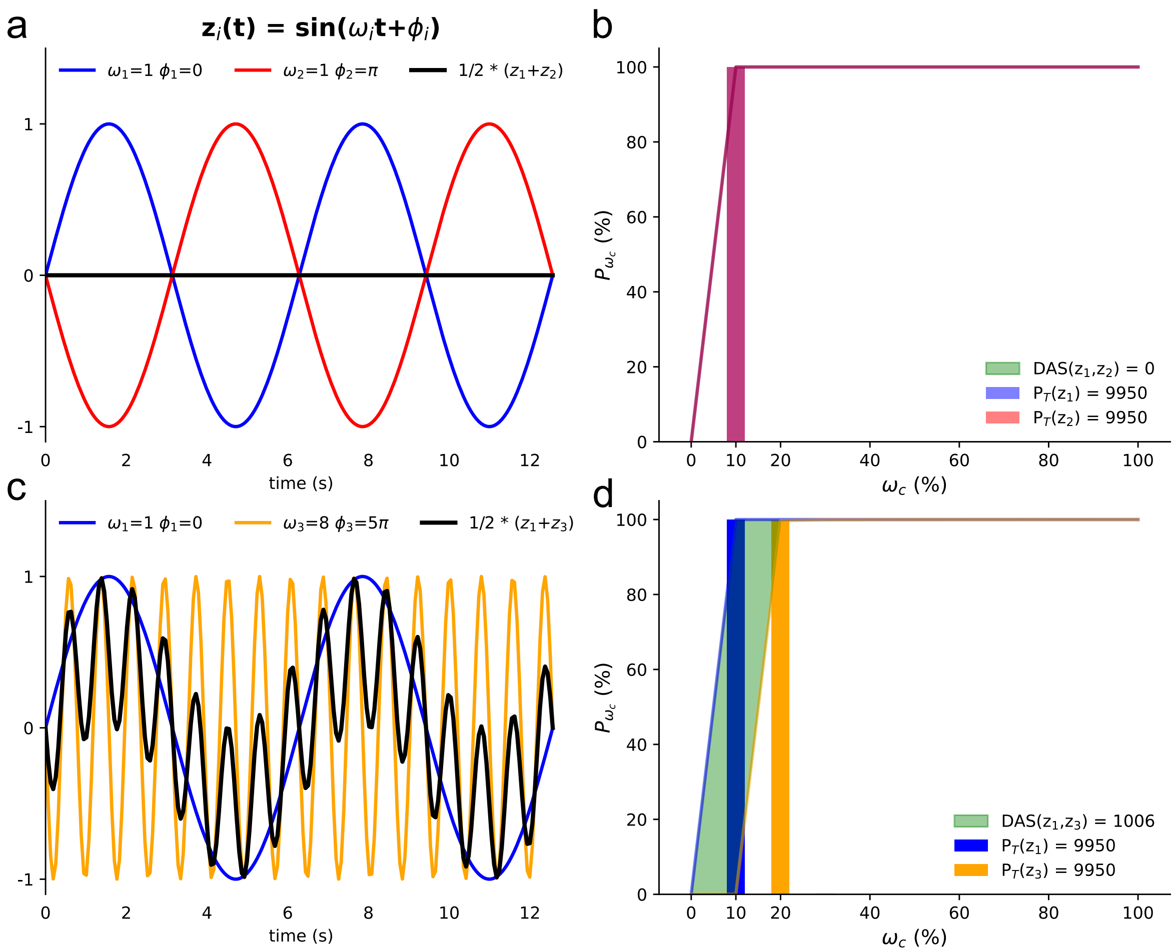

Fig. S1: DAS example of 3 sinusoidal signals. **a** Two sinusoidal signals with the same frequency and in complete dephasing. Their temporal correlation is exactly -1 and their mean is always zero and no coupled behavior can emerge. **b** The power spectrum, however, is identical (completely overlapping). Both signals, therefore, carry the same amount of power in the same frequency band. The lines represent the cumulative power distribution as defined in Eq. (3). In short, these two signals are indistinguishable since the dephasing might be a consequence of different starting time points. **c** Two sinusoidal signals with different frequencies of oscillations and arbitrary dephasing can carry the same power. The mean of both signals is now the composition of both frequencies. **d** The spectrum of frequencies is, nonetheless, very different. Conventional distance metrics would only capture differences in the information content, but the Dynamics Alteration Score (DAS) can distinguish which one of the signals displays faster or slower oscillations. The values of both the power and the DAS are entirely conditioned to the scale used (i.e., $DAS \in [0,1]$ if we had not defined the distributions as percentages).

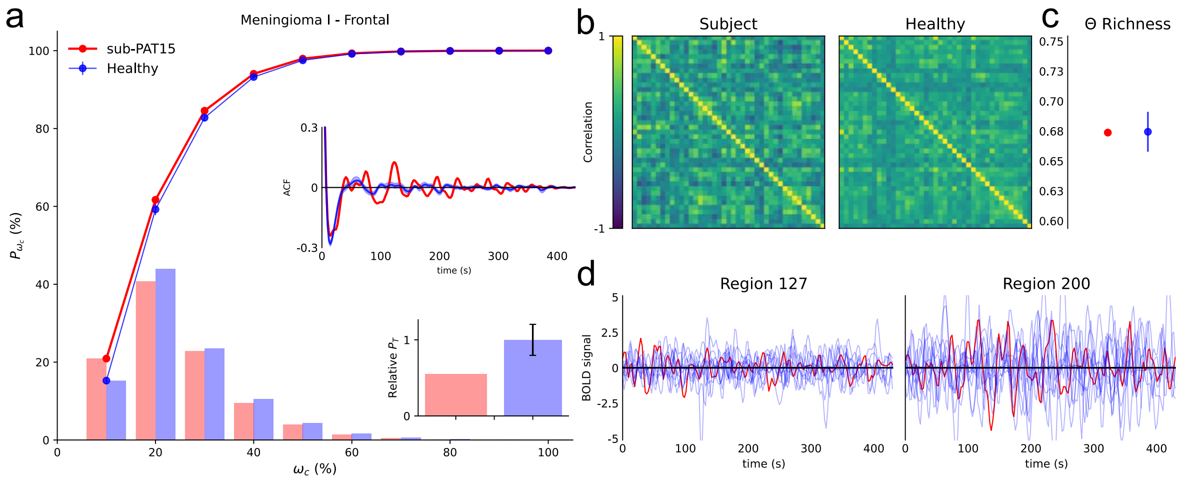

Fig. S2: sub-PAT15 DMN. **a** Cumulative power (lines), power distribution (bars), autocorrelation function (ACF), and total power relative to healthy subjects (histogram) for the patient (red) and mean of controls (blue) functional signals of the regions in the DMN. Error bars [mean ± SEM] indicate the results for the control population, while "*" codes for statistical significance. The relative power was obtained by averaging over subjects and DMN regions and taking healthy subjects as the baseline (i.e., equal to 1). **b** Functional DMN from the same patient and mean of control subjects (see Methods). **c** Functional complexity as measured by the distribution of correlations for the patient's network and the mean of control [mean ± SEM] (see Methods). **d** Functional signal of two randomly selected regions from the same patient (red) as opposed to all the control subjects (light blue). No apparent difference is found between raw time series across regions, subjects, and patients.

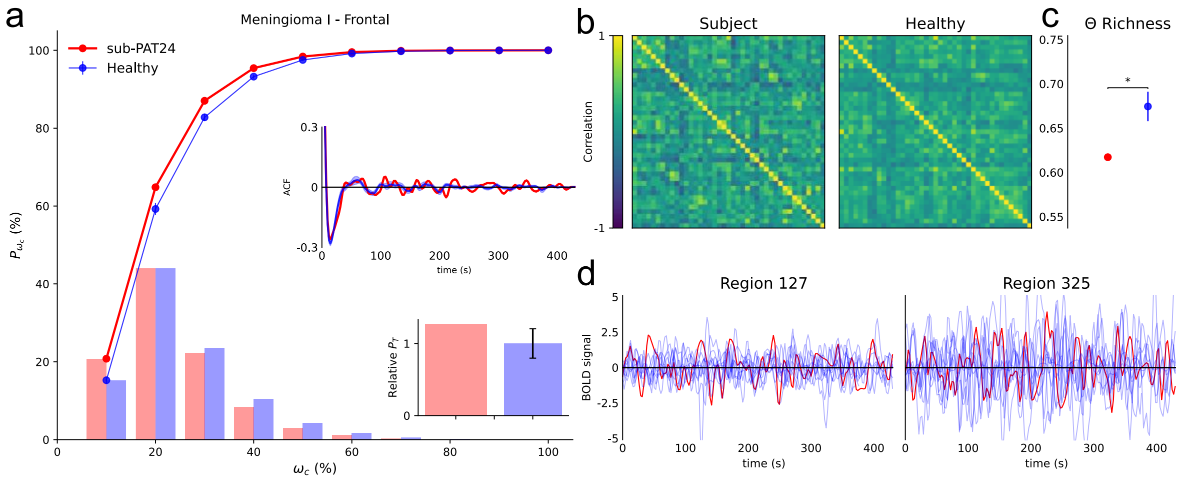

Fig. S3: sub-PAT24 DMN. **a** Cumulative power (lines), power distribution (bars), autocorrelation function (ACF), and total power relative to healthy subjects (histogram) for the patient (red) and mean of controls (blue) functional signals of the regions in the DMN. Error bars [mean ± SEM] indicate the results for the control population, while "*" codes for statistical significance. The relative power was obtained by averaging over subjects and DMN regions and taking healthy subjects as the baseline (i.e., equal to 1). **b** Functional DMN from the same patient and mean of control subjects (see Methods). **c** Functional complexity as measured by the distribution of correlations for the patient's network and the mean of control [mean ± SEM] (see Methods). **d** Functional signal of two randomly selected regions from the same patient (red) as opposed to all the control subjects (light blue). No apparent difference is found between raw time series across regions, subjects, and patients.

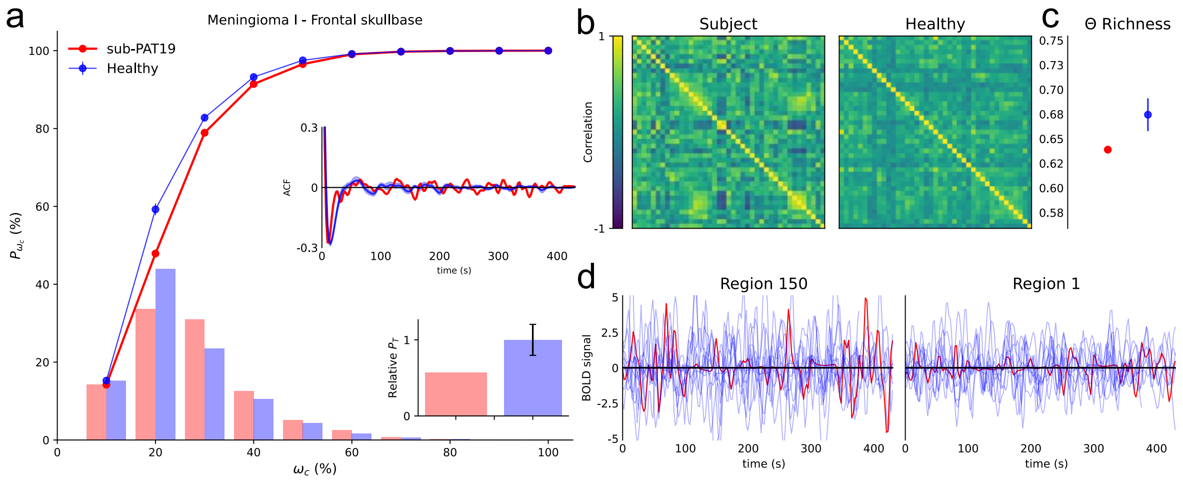

Fig. S4 sub-PAT19 DMN. **a** Cumulative power (lines), power distribution (bars), autocorrelation function (ACF), and total power relative to healthy subjects (histogram) for the patient (red) and mean of controls (blue) functional signals of the regions in the DMN. Error bars [mean ± SEM] indicate the results for the control population, while "*" codes for statistical significance. The relative power was obtained by averaging over subjects and DMN regions and taking healthy subjects as the baseline (i.e., equal to 1). **b** Functional DMN from the same patient and mean of control subjects (see Methods). **c** Functional complexity as measured by the distribution of correlations for the patient's network and the mean of control [mean ± SEM] (see Methods). **d** Functional signal of two randomly selected regions from the same patient (red) as opposed to all the control subjects (light blue). No apparent difference is found between raw time series across regions, subjects, and patients.

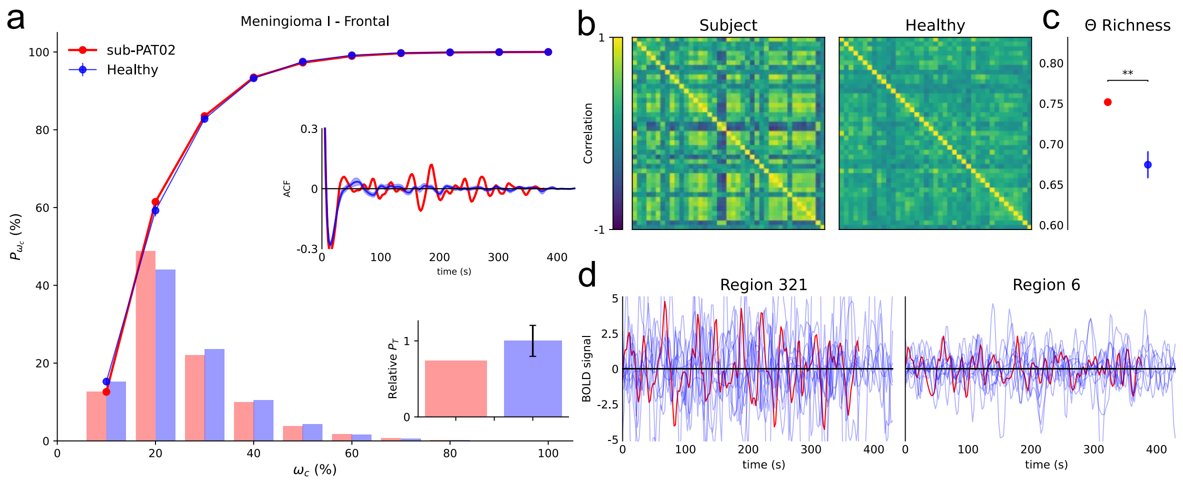

Fig. S5: sub-PAT02 DMN. **a** Cumulative power (lines), power distribution (bars), autocorrelation function (ACF), and total power relative to healthy subjects (histogram) for the patient (red) and mean of controls (blue) functional signals of the regions in the DMN. Error bars [mean ± SEM] indicate the results for the control population, while "*" codes for statistical significance. The relative power was obtained by averaging over subjects and DMN regions and taking healthy subjects as the baseline (i.e., equal to 1). **b** Functional DMN from the same patient and mean of control subjects (see Methods). **c** Functional complexity as measured by the distribution of correlations for the patient's network and the mean of control [mean ± SEM] (see Methods). **d** Functional signal of two randomly selected regions from the same patient (red) as opposed to all the control subjects (light blue). No apparent difference is found between raw time series across regions, subjects, and patients.

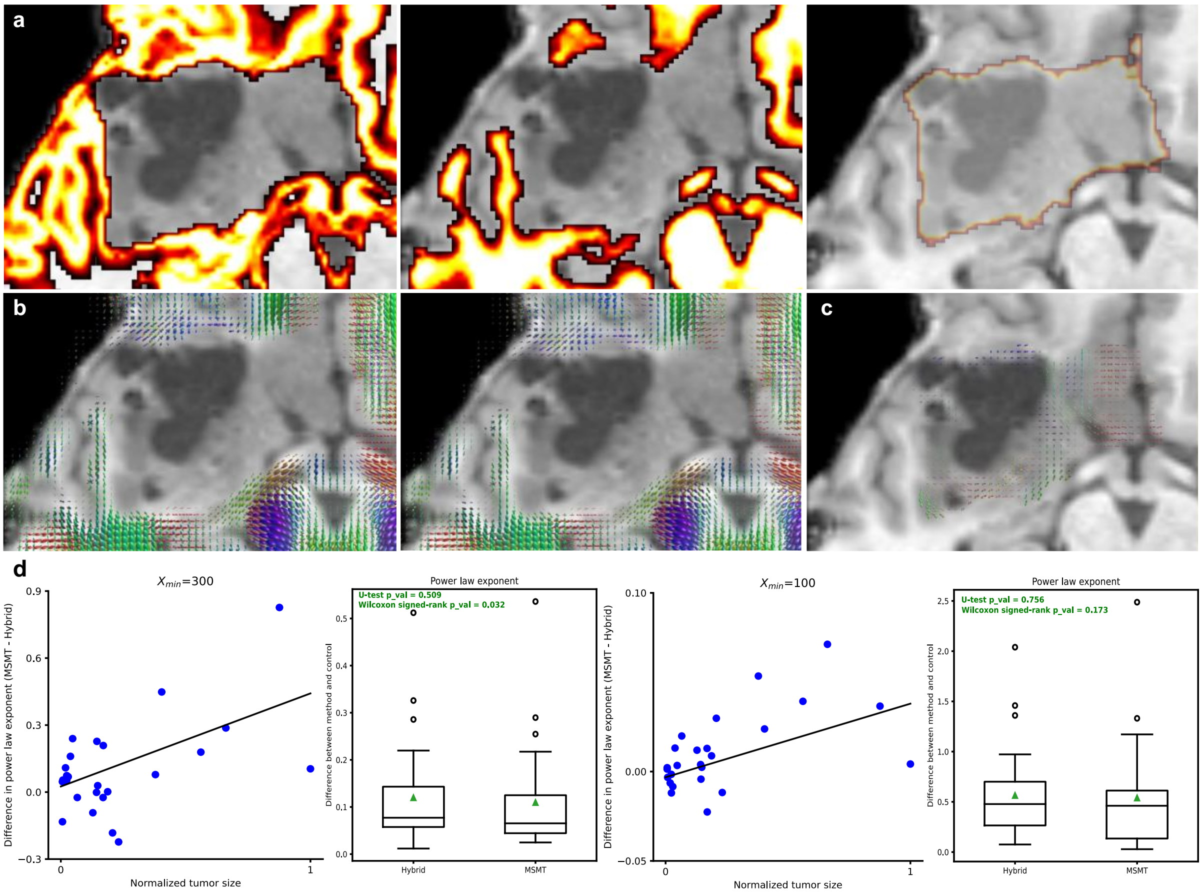

Fig. S6: Peritumoral anatomy and FODs. a Binary mask depicting the gray-matter (LEFT), white-matter (CENTER), and tumor (RIGHT) tissues of sub-PAT16. The tissue surrounding the lesion is automatically labeled as gray-matter thus imposing an anatomical barrier to regular fiber tracking algorithms. **b** Tumoral and peritumoral FODs reconstructed with the MSMT algorithm when explicitly masking the tumor (LEFT) and without masking the tumor (RIGHT). Even if the tumor is not masked, the algorithm damps the anisotropic component of the diffusion signal. **c** Tumoral and peritumoral FODs reconstructed with the SS3T algorithm applied only inside the lesion mask. Only this algorithm can recover the diffusion signal, which in this example, loosely maps the inflammation caused by the tumor. **d** Differences in the scale-freeness, as measured by the power law exponent, between the networks obtained with our pipeline and a canonical multi-shell multi-tissue approach (blue scatter plot) and between each pipeline and the healthy cohort (black boxplot). Green triangles depict the means, black horizontal lines depict the medians, and the boxes show the 1st and 3rd quartiles. Significance tests in green show the p-values for the differences between means. x_{min} refers to the minimum scaling range taken to fit the power law distribution. The higher the value, the more extremal values are considered.

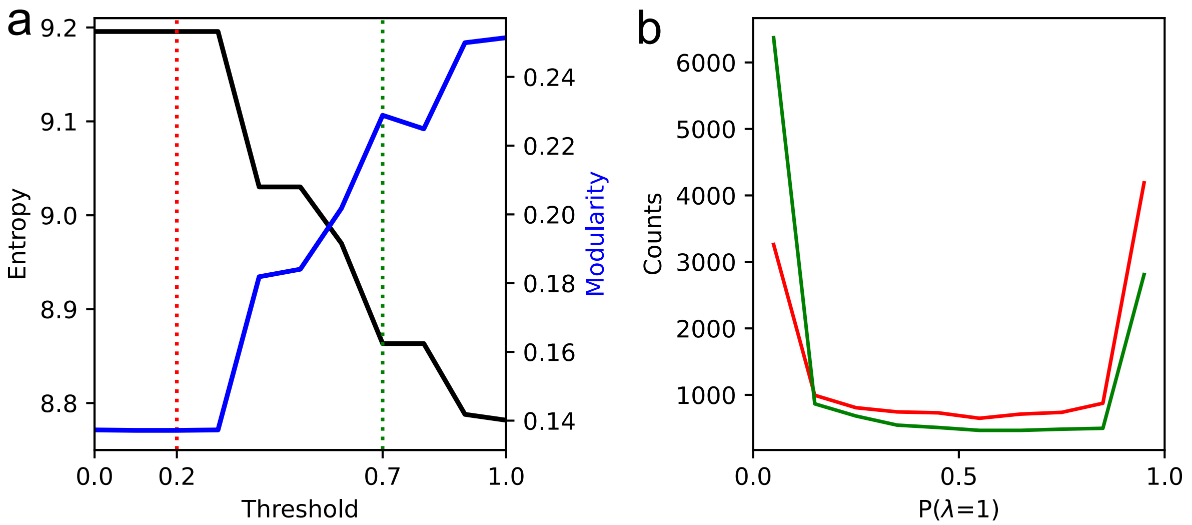

Fig. S7: Choosing the threshold for the Anatomical Prior. a Entropy and Modularity as a function of the selected threshold. These two properties were rather robust in certain intervals of the threshold value. Therefore, the choice of the threshold inside each one of these intervals was not critical to the performance of FCNET. Vertical dashed lines mark approximately the frontiers of the aforementioned intervals. **b** Number of occurrences of the variable described by Eq. S2 for each one of the thresholds marked previously. Higher thresholds deleted a considerably large number of possible connections limiting the generalization power of FCNET.

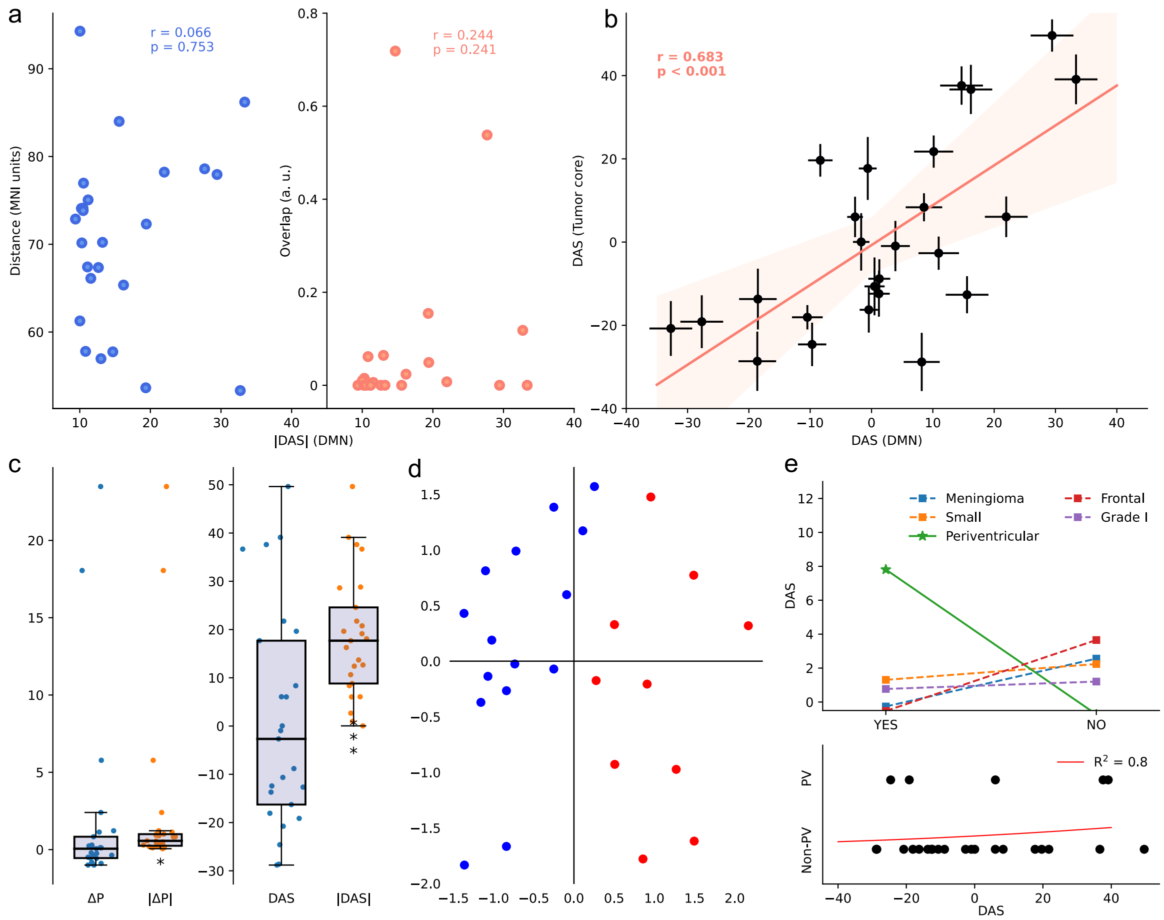

Fig. S8: DMN and tumor core BOLD signals. a Scatter plot showing the null correlation of DAS with distance and overlap between the tumor core and the DMN centroids. **b** A strong linear trend was found between alterations inside the patients' tumor core and alterations in the DMN as measured by the DAS. The orange line corresponds to the significant linear fit (two-sided exact test), and shaded areas mark the 95% confidence interval. **c** Differences in total power relative to healthy subjects (LEFT) and dynamics (RIGHT). Only the absolute values were significantly different from zero (one-side t-test and U-test). **d** Scatter plot of the two components found via Fast Independent Component Analysis applied to the DAS between patients and control subjects. The same two clusters (red, and blue) were consistently found. **e** TOP, Alteration in dynamics between different groups of tumors. BOTTOM, Logistic regression between DAS and periventricular (PV) tumor patients. Qualitative inspection reveals a tendency for periventricular tumors to display slower BOLD dynamics, although they were found to be non-significant presumably due to the small number of samples (N=5, p=0.33, one-sided U-test).

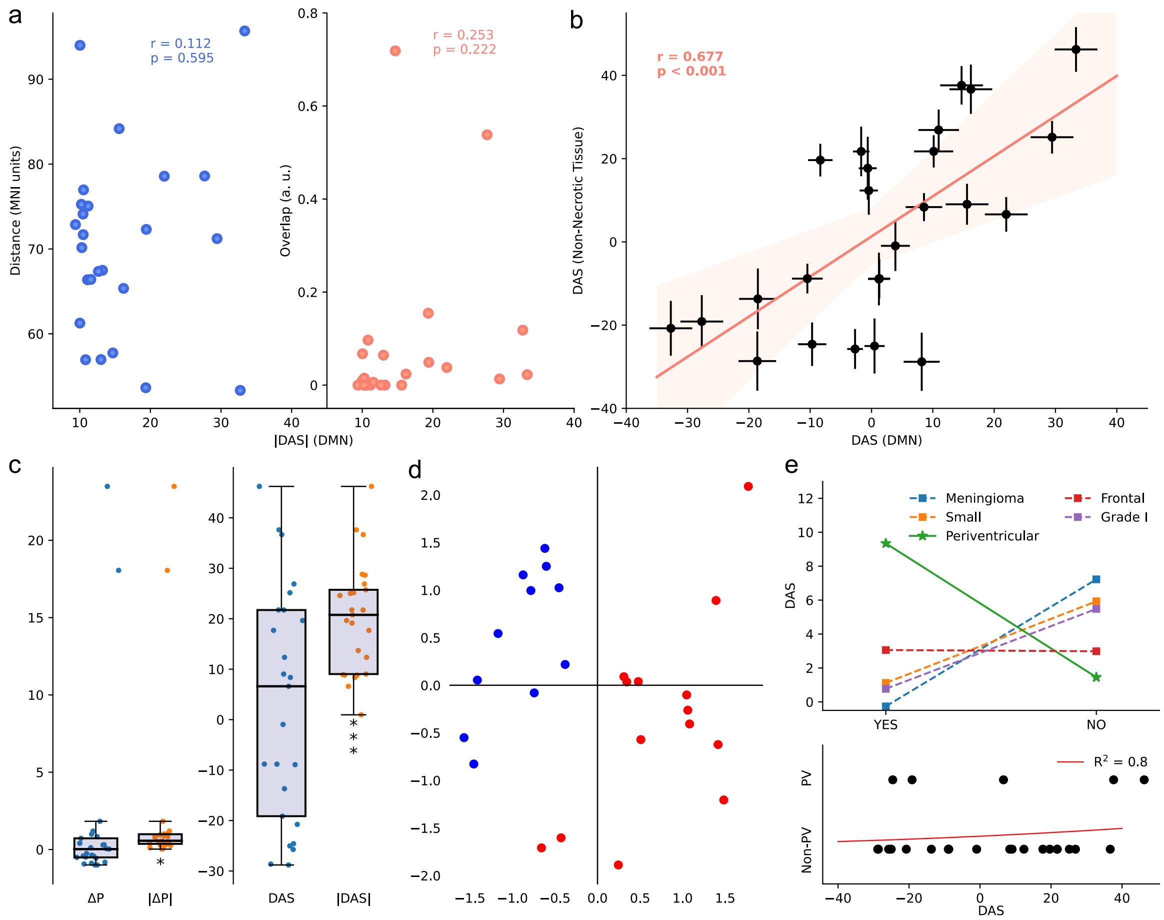

Fig. S9: DMN and non-necrotic tumor BOLD signals. a Scatter plot showing the null correlation of DAS with distance and overlap between the non-necrotic tissue and the DMN centroids. **b** A strong linear trend was found between alterations inside the patients' non-necrotic tissue and alterations in the DMN as measured by the DAS. The orange line corresponds to the significant linear fit (two-sided exact test), and shaded areas mark the 95% confidence interval. **c** Differences in total power relative to healthy subjects (LEFT) and dynamics (RIGHT). Only the absolute values were significantly different from zero (one-side t-test and U-test). **d** Scatter plot of the two components found via Fast Independent Component Analysis applied to the DAS between patients and control subjects. The same two clusters (red, and blue) were consistently found. **e** TOP, Alteration in dynamics between different groups of tumors. BOTTOM, Logistic regression between DAS and periventricular (PV) tumor patients. Qualitative inspection reveals a tendency for periventricular tumors to display slower BOLD dynamics, although they were found to be non-significant presumably due to the small number of samples (N=5, p=0.28, one-sided U-test).

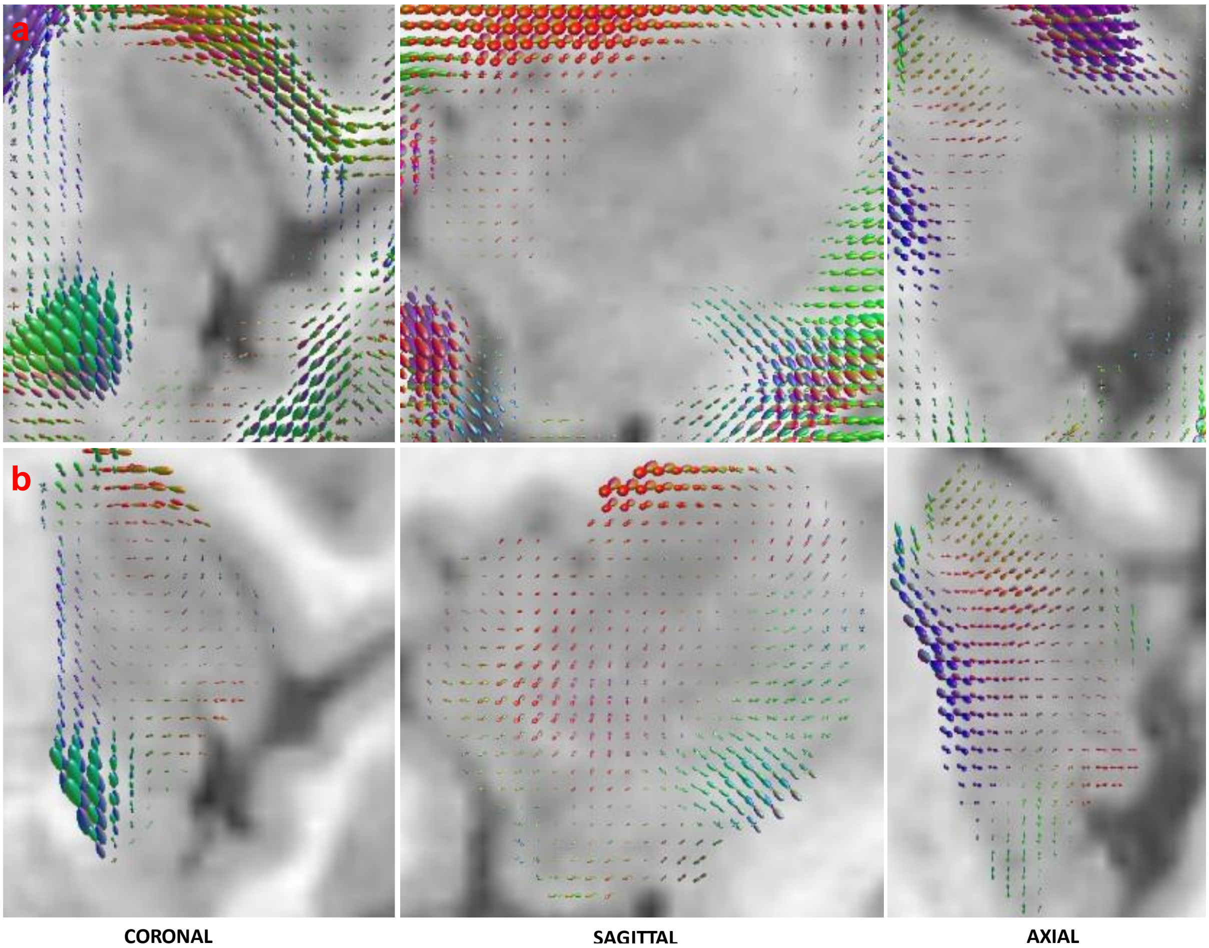

Fig. S10: Tumoral FODf reconstruction in sub-PAT05. Shown slices correspond to the intersection point (x,y,z) = (-36,1,-2) mm, or voxel (55,127,70) in MNI space. MRtrix3's viewer FODf display settings are detail 3, maximum order 8, and scale 5. **a** Using MSMT without masking the tumor. **b** Using SS3T only inside the lesion.

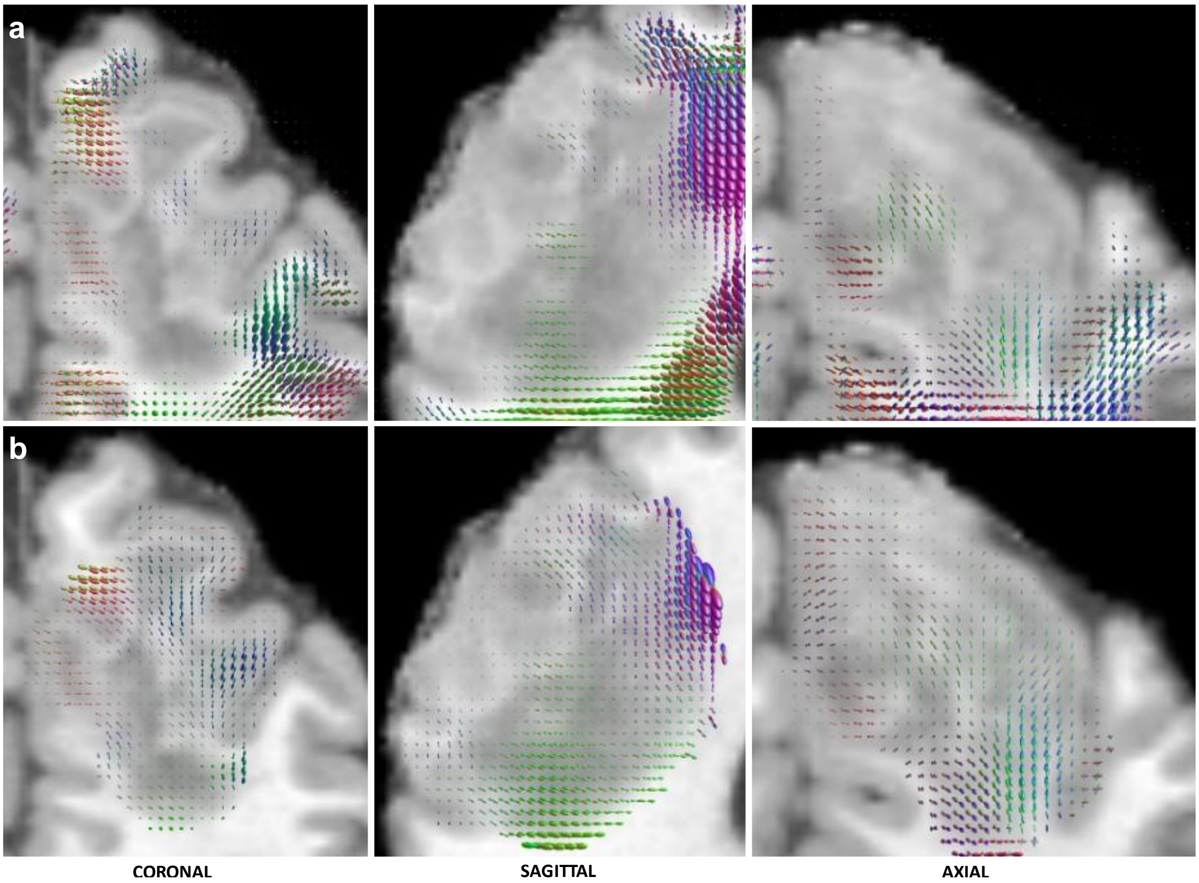

Fig. S11: Tumoral FODf reconstruction in sub-PAT29. Shown slices correspond to the intersection point (x,y,z) = (-15,43,-20) mm, or voxel (76,169,93) in MNI space. MRtrix3's viewer FODf display settings are detail 3, maximum order 8, and scale 5. **a** Using MSMT without masking the tumor. **b** Using SS3T only inside the lesion.

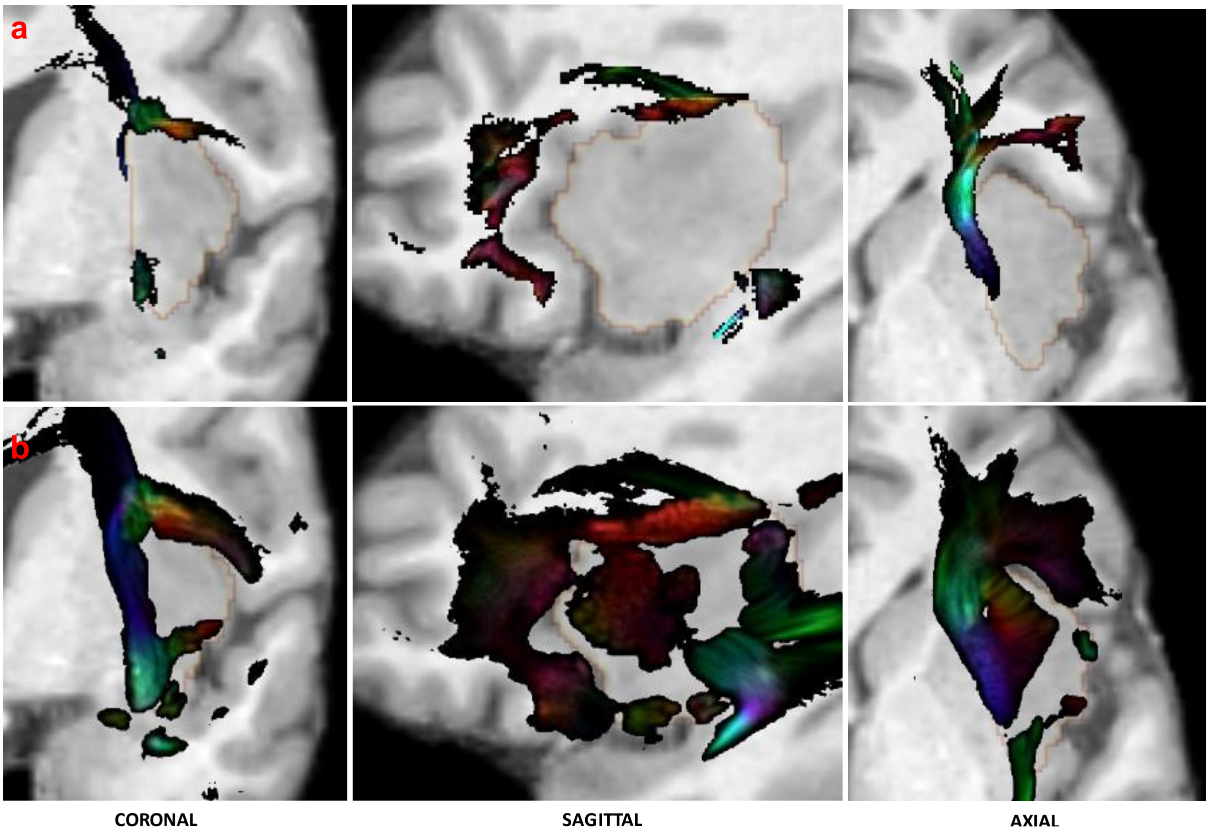

Fig. S12: Tumoral streamlines in sub-PAT05. Direction encoded color (DEC) track density maps shown correspond to point (x,y,z) = (-36,1,-2) mm, or voxel (55,127,70) in MNI space. **a** Using MSMT without masking the tumor. **b** Using SS3T only inside the lesion. DEC maps with an intensity threshold of [3,298.36] in **a** and [3,418.27] in **b.** Color bar of [10,180] in MRtrix3's viewer.

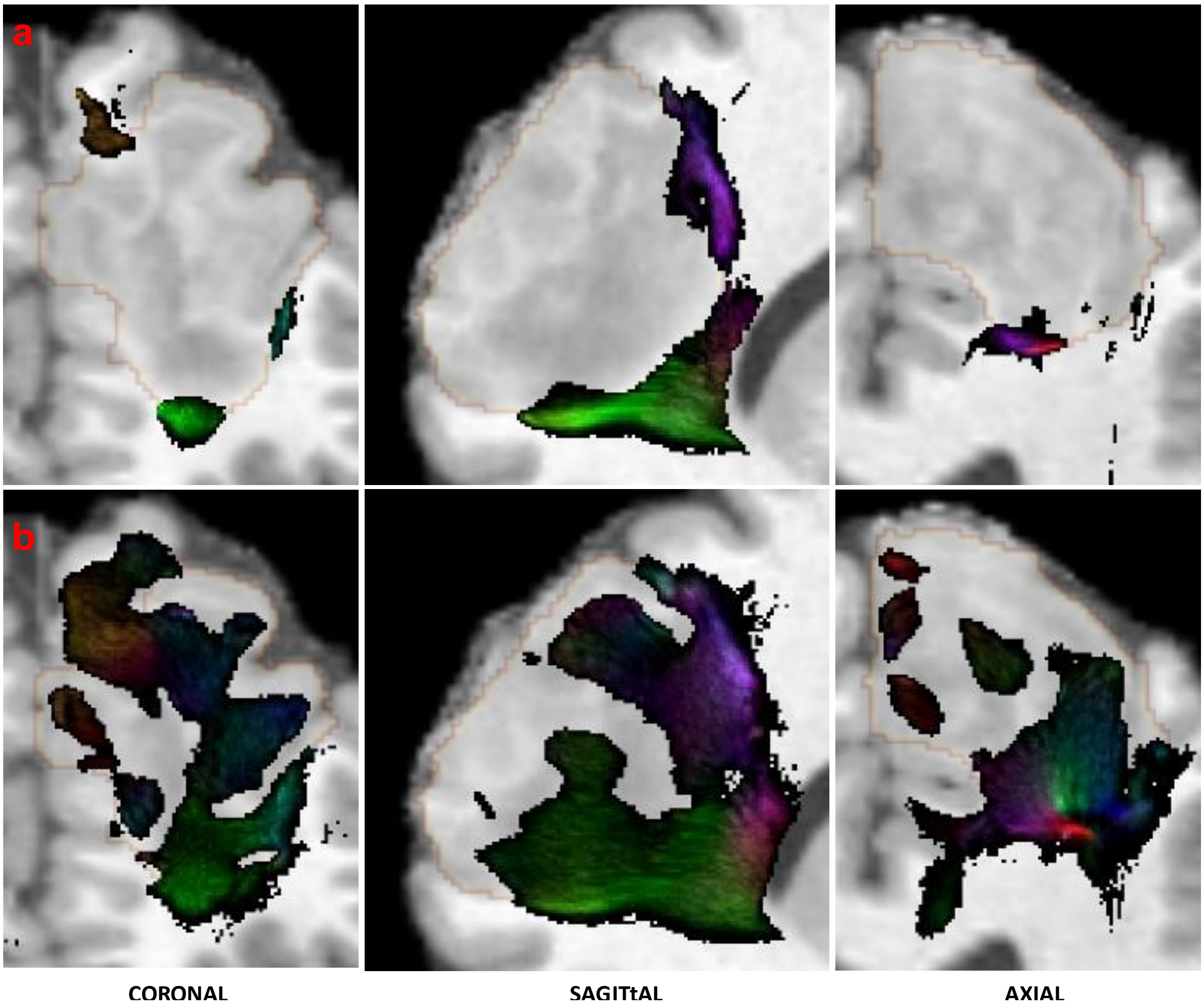

Fig. S13: Tumoral streamlines in sub-PAT29. Direction encoded color (DEC) track density maps shown correspond to point (x,y,z) = (-15,43,-20) mm, or voxel (76,169,93) in MNI space. **a** Using MSMT without masking the tumor. **b** Using SS3T only inside the lesion. DEC maps with an intensity threshold of [3,298.36] in **a** and [3,418.27] in **b.** Color bar of [10,180] in MRtrix3's viewer.

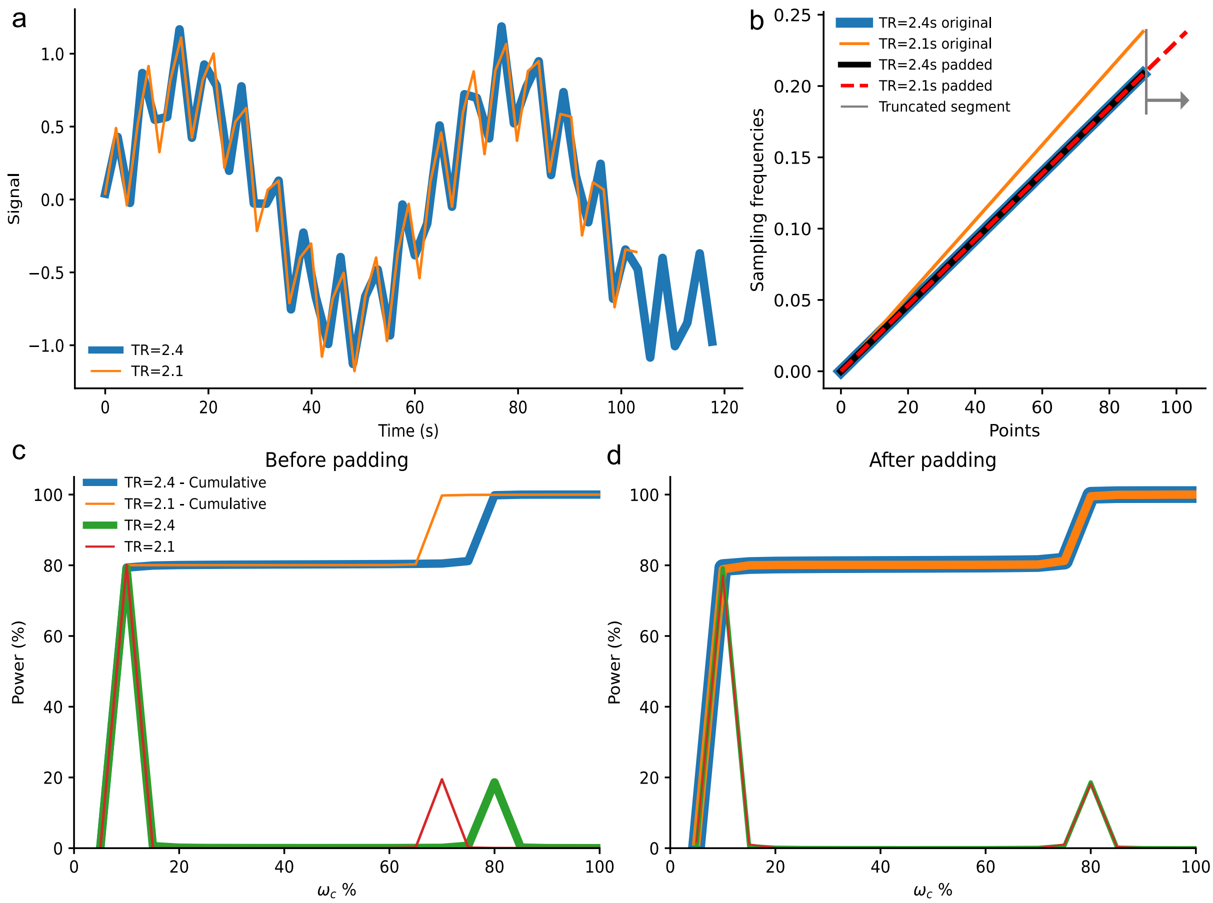

Fig. S14 Solving the TR inconsistency using Fourier analysis. a Two series sampled with different rates (TR=2400ms blue – TR=2100ms orange) while keeping the number of points constant at 180 points. As a result, the short TR series is shorter in time. The underlying signal consists of a combination of two sinusoidal signals, each one with different frequencies. Although the signal is the same, the sampled series differ slightly. Importantly, the TR mismatch in the real data is the same. **b** The discrete Fourier sampling frequencies for the original series in A and the series after adding zero-padding. Note how the black and red hyphened lines coincide after the truncation (frequencies to the right of the gray vertical line are discarded). **c, d** Power spectrum and cumulative power of the two series before (**c**) and after (**d**) zero-padding. Note that the cumulative power in the short unpadded series reaches 100% before the long series. This results in artificially altered DAS. After padding and truncation, these effects are successfully corrected.

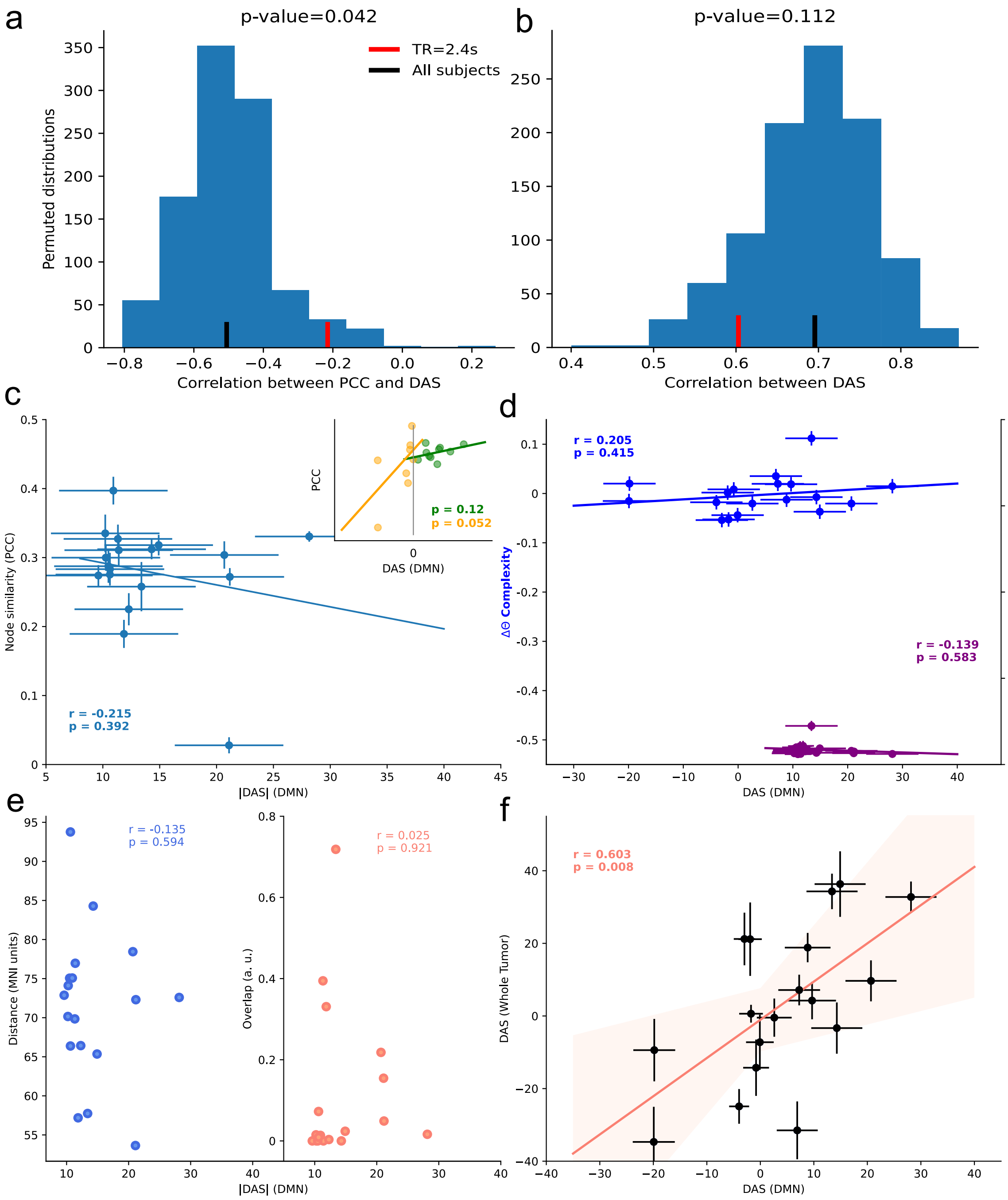

Fig. S15: Contribution of the Repetition Time to the reorganization of the network. a Null distribution of the correlation coefficients between the PCC and DAS of the DMN. **b** Null distribution of the correlation coefficients between the DAS of the DMN and the lesion. **c** Correlations between node similarity measured by the Pearson correlation coefficient and the Dynamics Alteration Score (DAS) of the Default Mode Network (DMN; only for subjects with TR=2.4s). The inset shows how alterations in the dynamics shape the organization of the network regardless of the direction of the displacement of the frequency spectrum. **d** Alterations in dynamics correlate with alterations in network complexity. Change in the complexity of the patients' DMN with respect to the healthy pool (Directionality ΔΘ and Magnitude |ΔΘ|) as a function of the DAS (only for subjects with TR=2.4s). **e** Scatter plot showing the null correlation of DAS with distance and overlap between the lesion and the DMN centroids (only for subjects with TR=2.4s). **f** A strong linear trend was found between alterations inside the patients' tumor and alterations in the DMN as measured by DAS (only for subjects with TR=2.4s). The orange line corresponds to the significant linear fit (two-sided exact test), and the shaded areas mark the 95% confidence interval.
