# Supplementary material for: Functional and structural reorganization in brain tumors: a machine learning approach using desynchronized functional oscillations
Source: Commun Biol. 2024 Apr 6;7:419. doi: 10.1038/s42003-024-06119-3 (PMC10998892; doi:10.1038/s42003-024-06119-3)
Supplement: Supplementary file 3 — Reporting Summary [file 42003_2024_6119_MOESM3_ESM.pdf]

nature portfolio

Corresponding author(s): DBPR COMMSBIO-22-4195C

Last updated by author(s): Mar 8, 2024

Reporting Summary

Nature Portfolio wishes to improve the reproducibility of the work that we publish. This form provides structure for consistency and transparency in reporting. For further information on Nature Portfolio policies, see our Editorial Policies and the Editorial Policy Checklist.

Statistics

For all statistical analyses, confirm that the following items are present in the figure legend, table legend, main text, or Methods section.

n/a	Confirmed	
☐	☒	The exact sample size (*n*) for each experimental group/condition, given as a discrete number and unit of measurement
☐	☒	A statement on whether measurements were taken from distinct samples or whether the same sample was measured repeatedly
☐	☒	The statistical test(s) used AND whether they are one- or two-sided<br>*Only common tests should be described solely by name; describe more complex techniques in the Methods section.*
☒	☐	A description of all covariates tested
☐	☒	A description of any assumptions or corrections, such as tests of normality and adjustment for multiple comparisons
☐	☒	A full description of the statistical parameters including central tendency (e.g. means) or other basic estimates (e.g. regression coefficient) AND variation (e.g. standard deviation) or associated estimates of uncertainty (e.g. confidence intervals)
☐	☒	For null hypothesis testing, the test statistic (e.g. *F*, *t*, *r*) with confidence intervals, effect sizes, degrees of freedom and *P* value noted<br>*Give P values as exact values whenever suitable.*
☐	☒	For Bayesian analysis, information on the choice of priors and Markov chain Monte Carlo settings
☒	☐	For hierarchical and complex designs, identification of the appropriate level for tests and full reporting of outcomes
☐	☒	Estimates of effect sizes (e.g. Cohen's *d*, Pearson's *r*), indicating how they were calculated

Our web collection on statistics for biologists contains articles on many of the points above.

Software and code

Policy information about availability of computer code

Data collection	Anatomical, diffusion and functional MRI were collected using a Siemens 3T Magnetom Trio MRI scanner with a 32-channel head coil. For more details see Aerts, et al. (2022).
Data analysis	We used fmriprep, FSL, MRtrix3, DIPY, ANTS, NetworkX and Python for analysis of MRIs and brain networks.

For manuscripts utilizing custom algorithms or software that are central to the research but not yet described in published literature, software must be made available to editors and reviewers. We strongly encourage code deposition in a community repository (e.g. GitHub). See the Nature Portfolio guidelines for submitting code & software for further information.

Data

Policy information about availability of data

All manuscripts must include a data availability statement. This statement should provide the following information, where applicable:

- Accession codes, unique identifiers, or web links for publicly available datasets
- A description of any restrictions on data availability
- For clinical datasets or third party data, please ensure that the statement adheres to our policy

The original data is publicly available at OpenNeuro and published in Open Access Journal (H. Aerts, N. Colenbier, H. Almgren, T. Dhollander, J. R. Daparte, K. Clauw, A. Johri, J. Meier, J. Palmer, M. Schirner and others, “Pre-and post-surgery brain tumor multimodal magnetic resonance imaging data optimized for large scale computational modelling,” Scientific Data, vol. 9, p. 1–10, 2022.)

Human research participants

Policy information about studies involving human research participants and Sex and Gender in Research.

Reporting on sex and gender	Participants included 19 females and 17 males (considering biological attribute). Nonetheless, the analysis performed did not segregate according to sex nor gender, hence no conclusions regarding these two groups were stated.
Population characteristics	Out of the 36 subjects that agreed to take part in the pre-surgery session (11 healthy [58.6 ± 10.6 years], 14 meningioma [60.4 ± 12.3 years] and 11 glioma [47.5 ± 11.3 years]), 28 were scanned after a period spanning from 6 to 12 months in the post-surgery session (10 healthy [59.6 ± 10.3 years], 12 meningioma [57.9 ± 11.0 years] and 7 glioma [50.7± 11.7 years]). As a result, 19 pre- and post-surgery pairs of structural connectomes were usable as training and testing data. All brain tumors were classified as grade I, II, and III according to the World Health Organization.
Recruitment	Patients were recruited at Ghent University Hospital (Belgium) between May 2015 and October 2017. Patients were eligible if they (1) were at least 18 years old, (2) had a supratentorial meningioma (WHO grade I or II) or glioma (WHO grade II or III) brain tumor, (3) were able to complete neuropsychological testing, and (4) were medically approved to undergo MRI investigation. Partners were also asked to participate in the study to constitute a group of control subjects that suffer from emotional distress comparable to that of the patients.
Ethics oversight	All participants received detailed study information and gave written informed consent before study enrollment. This research was approved by the Ethics Committee at Ghent University Hospital.

Note that full information on the approval of the study protocol must also be provided in the manuscript.

Field-specific reporting

Please select the one below that is the best fit for your research. If you are not sure, read the appropriate sections before making your selection.

☒ Life sciences ☐ Behavioural & social sciences ☐ Ecological, evolutionary & environmental sciences

For a reference copy of the document with all sections, see nature.com/documents/nr-reporting-summary-flat.pdf

Life sciences study design

All studies must disclose on these points even when the disclosure is negative.

Sample size	A total of 36 subjects. 14 meningioma, 11 glioma and 11 controls.
Data exclusions	From the original data available in Aerts, et al. (2022), no exclusions were done.
Replication	Replicability of this study was not possible due to lack of external validation dataset. The uniqueness of the paired pre- and post-surgery samples makes explicit replication difficult. Yet, this may be tackled in future studies given the appropiate data. Nonetheless, full transparency in the manuscript is emphasized and facilitated by publicly available codes.
Randomization	Randomization was not applicable since the experimental groups were determined solely based on histological considerations. However, for certain statistical analyses, permutation tests were carried out.
Blinding	Blinding was not applicable.

Reporting for specific materials, systems and methods

We require information from authors about some types of materials, experimental systems and methods used in many studies. Here, indicate whether each material, system or method listed is relevant to your study. If you are not sure if a list item applies to your research, read the appropriate section before selecting a response.

Materials & experimental systems

n/a	Involved in the study
☒	☐ Antibodies
☒	☐ Eukaryotic cell lines
☒	☐ Palaeontology and archaeology
☒	☐ Animals and other organisms
☒	☐ Clinical data
☒	☐ Dual use research of concern

Methods

n/a	Involved in the study
☒	☐ ChIP-seq
☒	☐ Flow cytometry
☐	☒ MRI-based neuroimaging

Magnetic resonance imaging

Experimental design

Design type	Anatomical/Structural, diffusion and resting-state 3 Tesla MRI
Design specifications	The design is described in Aerts, et al. (2022). T1-MPRAGE MRI, HARDI multi-shell diffusion MRI and resting-state functional MRI data was acquired in 2 sessions separated by a time span of approximately 12 months (pre- and post-surgery).
Behavioral performance measures	For the present study, behavioral recordings are not applicable. Details for the original recordings are thouroughly explained in Aerts, et al. (2022).

Acquisition

Imaging type(s)	Structural, Diffusion and Functional magnetic resonance imaging
Field strength	3 Tesla
Sequence & imaging parameters	T1-MPRAGE anatomical scan: 160 slices, TR = 1750 ms, TE = 4.18 ms, field of view = 256 mm, flip angle = 9o, voxel size 1 x 1 x 1 mm3, acquisition time of 4:05 min.<br><br>Multi-shell HARDI diffusion MRI acquisition: 60 slices, TR = 8700 ms, TE = 110 ms, field of view = 240 mm, voxel size 2.5 x 2.5 x 2.5 mm3, acquisition time of 15:14 min, 101-102 directions b = 0, 700, 1200, 2800 s/mm2; together with two reversed phase-encoding b = 0 s/mm2 blips for the purpose of correcting susceptibility-induced distortions.<br><br>Resting-state functional echo-planar imaging data were obtained: 42 slices, TR = 2100 ms, TE = 27 ms, field of view = 192 mm, flip angle = 90o, voxel size 3 x 3 x 3 mm3, acquisition time of 6:24 min. The TR was accidentally changed to 2400 ms after 4 control subjects, 5 meningioma patients and 2 glioma patients were scanned changing the times of acquisition to 7:19 min.
Area of acquisition	Whole brain scan

Diffusion MRI ☒ Used ☐ Not used

Parameters: See above (Sequence & imaging parameters)

Preprocessing

Preprocessing software	A more detailed description can be found in the main manuscript.<br>Preprocessing of anatomical scans: FSL from Smith, et al. (2004) and ANTS from Avants, et al. (2009).<br>Preprocessing of diffusion scans: MRtrix3 from Tournier, et al. (2019) and DIPY from Garifallidis, et al. (2014).<br>Preprocessing of functional scans: fmriprep 20.2.2 from Esteban, et al. (2018).
Normalization	Diffusion and anatomical images were linearly registered to MNI (Montreal Neurological Space) standard space using FSL 'flirt'. fMRI images were registered according to fmriprep internal workflows.
Normalization template	Automated Anatomical Labelling Atlas 3 (AAL3) reference volume in MNI space and Gordon cortical parcellation.
Noise and artifact removal	For anatomical images bias field correction with N4BiasField from ANTS.<br><br>For diffusion images, bias field with N4BiasField from ANTS,s usceptibility induced distortions with FSL topup and eddy currents removal with FSL eddycuda.<br><br>For functional images, 36P (Satterwaite, et al. 2013; Ciric et al,2017): 6 MOTION + CSF and WM signals + global signal; their derivatives, their squares, and their squared derivative; linear and polinomial trend regression; band-pass filtering. The denoising was performed by running XCP-D (Ciric, et al. 2018).

Volume censoring	Temporal Censoring using a Framewise Displacement (FD) of 0.3 mm

Statistical modeling & inference

Model type and settings	Univariate linear regression and multivariate non-linear regression (fully connected artificial neural network).
Effect(s) tested	For the functional data the hipothesis was that the presence of the tumor would significantly alter the power spectrum of the fMRIsignal causing global reorganization of functional networks. For the structural networks, built from diffusion MRI, we tested the predictability of post-surgery brain networks from pre-operative scans.

Specify type of analysis: ☐ Whole brain ☒ ROI-based ☐ Both

Anatomical location(s)	ROIs defined according to the AAL3 parcellation (Rolls, et al. 2020) and the (Gordon cortical parcellation (Gordon, et al. 2016)
Statistic type for inference (See Eklund et al. 2016)	Parametric and nonparametric statistical tests were conducted.
Correction	No correction for multiple hypotheses testing was applied nor needed.

Models & analysis

n/a	Involved in the study
☐	☒ Functional and/or effective connectivity
☐	☒ Graph analysis
☐	☒ Multivariate modeling or predictive analysis

Functional and/or effective connectivity	Functional connectivity measured by pearson correlation
Graph analysis	Networks consisted of weighted connections. Numerical similarity based on node-wise pearson correlation; dynamical richness based on Zamora-Lopez, et al. (2016); weight-degree distribution.
Multivariate modeling and predictive analysis	A fully connected artificial neural network was trained to predict how structural brain networks estimated from diffusion MRI will react and adapt after brain tumor removal (i.e., post-surgery). To avoid overfitting, data was split into train-validation-test sets. To test for generalization a 1-fold cross validation schema was adopted. Epochs were kept at 100 and the mean squared error was the function to optimize. Weight degree distributions were tested to assess topological accuracy of generated networks.
